# Correction: Analysis of epidemiological association patterns of serum thyrotropin by combining random forests and Bayesian networks

**DOI:** 10.1371/journal.pone.0294489

**Published:** 2023-11-10

**Authors:** Ann-Kristin Becker, Till Ittermann, Markus Dörr, Stephan B. Felix, Matthias Nauck, Alexander Teumer, Uwe Völker, Henry Völzke, Lars Kaderali, Neetika Nath

There are errors in the Funding section. The correct Funding statement is: SHIP is part of the Community Medicine Research network of the University of Greifswald, Germany, which is funded by the Federal Ministry of Education and Research (grants no. 01ZZ9603, 01ZZ0103, and 01ZZ0403), the Ministry of Cultural Affairs as well as the Social Ministry of the Federal State of Mecklenburg-West Pomerania, and the network ’Greifswald Approach to Individualized Medicine (GANI_MED)’ funded by the Federal Ministry of Education and Research (grant 03IS2061A). Genome-wide data have been supported by the Federal Ministry of Education and Research (grant no. 03ZIK012) and a joint grant from Siemens Healthineers, Erlangen, Germany and the Federal State of Mecklenburg- West Pomerania. The University of Greifswald is a member of the Caché Campus program of the InterSystems GmbH. This work was further supported by the project Superthyreose, funded by the German “Innovationsfonds des Gemeinsamen Bundesausschusses” (grant no. VSF2_2019–167) AKB received funding from the BMBF (LiSyM, grant number 031L0032) and gratefully acknowledges an add-on-fellowship from the Joachim Herz Stiftung. LK acknowledges funding from the European Union (EuCanShare, grant 825903), as well as the State of Lower Saxony and the Volkswagenstiftung (Indira, grant number ZN3437). We acknowledge support for the Article Processing Charge from the DFG and the Open Access Publication Fund of the University of Greifswald. The funders had no influence on study design, data analysis, study interpretation, decision to publish, and writing of the manuscript.
