# Supplementary figures and images for: Analysis of epidemiological association patterns of serum thyrotropin by combining random forests and Bayesian networks
Source: PLoS One. 2022 Jul 21;17(7):e0271610. doi: 10.1371/journal.pone.0271610 (PMC9302835; doi:10.1371/journal.pone.0271610)

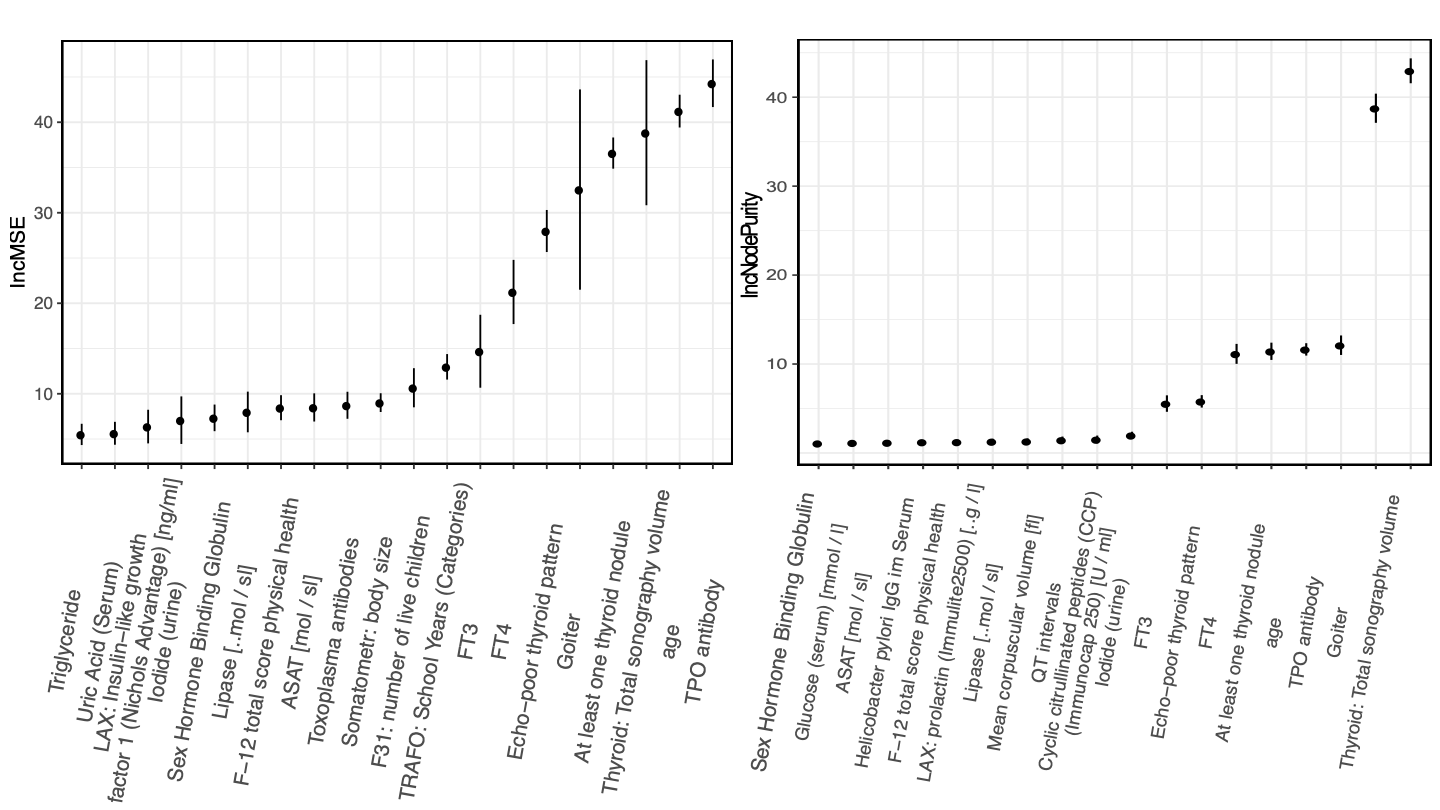

Supplement: S1 Fig — Based on A) incremental mean square error (IncMSE) and B) Node purity, the highest importance scores were found for age, FT3, FT4, anti-TPO antibodies, goiter, thyroid nodules, and thyroid hypoechogenicity in sonography. The important features were extracted from random forest based on the selected parameter nodesize of 15, and maxnodes of 15. (TIF) [file pone.0271610.s001.tif]

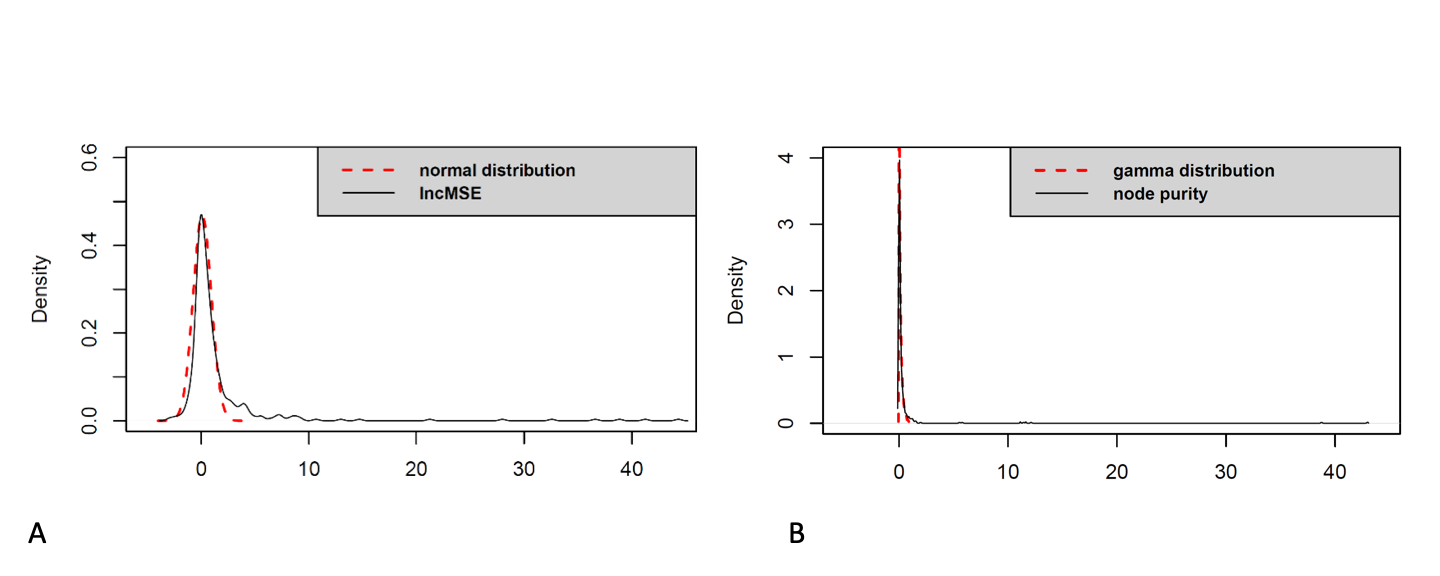

Supplement: S2 Fig — A statistical mixture model was used, the component around zero (red dashed lines) was modeled as A) a normal distribution for IncMSE (mean 0.1, standard deviation) B) a Gamma distribution for node purity (shape parameter p = 0.62 and scale parameter b = 4.65). Features were identified as relevant if they had feature importance larger than the respective 0.999-quantile. (TIF) [file pone.0271610.s002.tif]

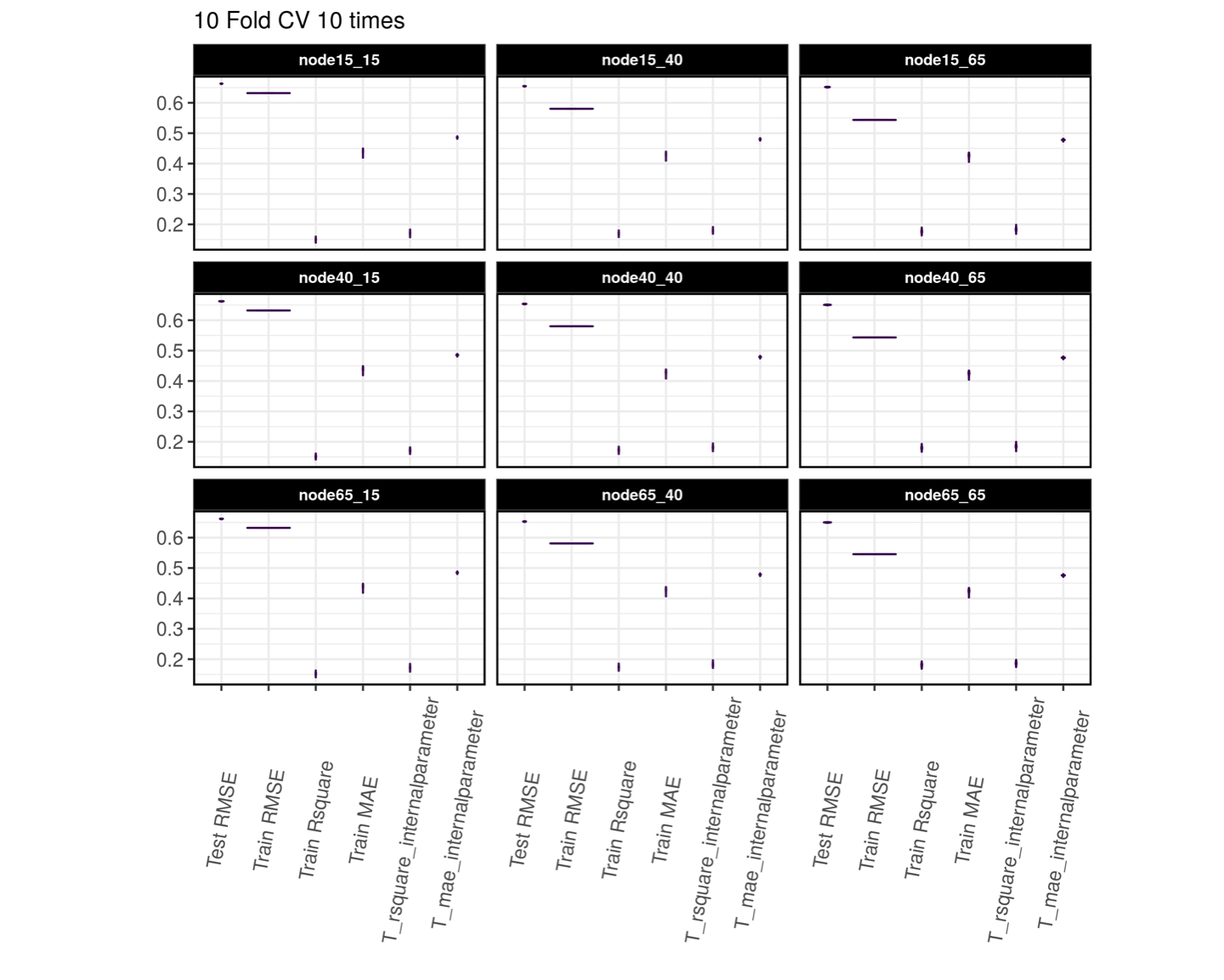

Supplement: S3 Fig — Prediction results from the grid-based hyperparameter optimization of the random forest model using 10-fold nested cross-validation. Rows show results for varying values of the parameter nodesize (tested values: 15, 40, 65), and columns show results for varying values of the maximal number of terminal nodes (maxnodes, tested values: 15, 40, 65). The parameter mtry was optimized separately and set to 5. (TIF) [file pone.0271610.s003.tif]
