# Supplementary material for: Analysis of epidemiological association patterns of serum thyrotropin by combining random forests and Bayesian networks
Source: PLoS One. 2022 Jul 21;17(7):e0271610. doi: 10.1371/journal.pone.0271610 (PMC9302835; doi:10.1371/journal.pone.0271610)
Supplement: Attachment — Submitted filename: ResponseToReviewers.pdf [file pone.0271610.s009.pdf]

Institute of Bioinformatics · Felix-Hausdorff-Str.8 · 17475 Greifswald

Institute of Bioinformatics
University Medicine Greifswald
Director: Prof. Dr. Lars Kaderali

Tel.: +49 3834 86-5441
E-Mail: lars.kaderali@uni-greifswald.de

June 29, 2022

Response to Reviewers

The responses to the concerns raised by reviewers are below and are color-coded as follows:

- Comments from editors or reviewers are shown as text;
- Our responses are shown as text.

Comments of Reviewer #1:

The manuscript "Analysis of epidemiological association patterns of serum Thyrotropin by combining random forests and Bayesian networks " presents a valuable and interesting approach to how two techniques can be combined to create predictive models. The work has been carried out with care and is presented in sufficient detail. The manuscript has been reviewed before and thereafter transferred to PlosONE. Reviser's comments, re-comments by the authors and corrections in the text are contained in the submission. The editor comment on rejection was: "While the reviewers do not find any major technical flaws that would preclude publication of the work, they also mark on the lack of depth of the manuscript and potential practical relevance".
I agree with the editors statement, that there are no major technical flaws that would preclude publication. I disagree with the statement of "lack of depth of the manuscript and potential practical relevance".

Main critics was given by previous reviewer #1 in two points:

a) *He/she is missing a comparison with other existing methods … making this research of interests for the bioinformatics and ML community.*

   The reviewer's request is understandable but hardly realizable. There a plenty of ways how build reliable prediction models, especially if techniques are combines. Further, the generalization of any comparison lacks the scenarios simulated for it. The authors already present a simulation, showing that their approach picks out necessary features for prediction. They intensively applied cross validation, a well-accepted technique for robust model building.

b) *The reviewer implicitly focus on ML models.*

UNIVERSITÄTSMEDIZIN GREIFSWALD · KÖRPERSCHAFT DES ÖFFENTLICHEN RECHTS
VORSTAND: Prof. Dr. Claus-Dieter Heidecke (ÄV) Prof. Dr. Karlhans Endlich (WV) · Marie le Claire (KV)
AUFSICHTSRATSVORSITZENDER: Sebastian Schröder
Fleischmannstraße 8 · 17475 Greifswald · Tel.: +49 (0) 3834 86 0 · www.medizin.uni-greifswald.de

I understand the author's aim of their approach is in improving prediction by combining methods, not stating that their approach is universal best. It goes without saying, that the concept can also be used if, for example, the Bayesian Network is replaced by a ML-prediction models.

c) *Another point of critics was given by previous reviewer #2 regarding the practical value of predicting TSH levels from other blood-based variables, instead of directly measuring from blood.*

   A possible application of the presented results could be follows: A measured TSH level that exceeds the predicted value may raise medical concerns about finding the cause. Or: A predicted high TSH value should be verified by direct measurement. The application of the proposed results is not the scope of the manuscript.

   All in all, I recommend the manuscript for publication without the need for further revision.

Author response: We thank the reviewer for the clear summary of our work and for the encouraging words. We agree to the explanations that are given in response to the comments of the previous reviewer. We chose random forests due to their well-known efficient handling of heterogeneous data structures, which is a typical scenario in a clinical and epidemiological dataset such as the Study of Health in Pomerania (SHIP). To enhance the interpretability of the model, BNs were included into our workflow as they are highly flexible, yet clearly interpretable. We agree that the proposed workflow could be transferred to other ML-prediction models and with the suggested clinical application scenario.

Comments of Reviewer #2:

1. *The algorithm as outlined is reasonable but the approach is not entirely novel. The authors perform what's known as Markov Blanket feature selection before modeling the data with a Bayes Net. Indeed, it is somewhat unusual to perform BN fitting without prior variable selection on a large-enough dataset. For a similar example where SVMs are used to perform feature selection see, for example:*

   Shen et al, Markov Blanket Feature Selection for Support Vector Machines, AAAI, 2008

   and for the theoretical justification of this type of a first-pass filter see this foundational paper:

   Koller and Sahami, Toward Optimal Feature Selection, ICML, 1996

   This comment should be interpreted as a suggestion for a useful reference or two, which would help build the context for the work, but does not invalidate the technical accuracy or practical utility of the authors' approach. Please consider this as a request for a very minor revision.

Author response: We appreciate the reviewer's feedback and agree that the references can add context to our modeling approach. Markov Blanket Feature Selection usually combines two modelling steps (learning the Markov Blanket and training a predictive model) which can be seen as similar to our described approach. However, as you write

UNIVERSITÄTSMEDIZIN GREIFSWALD · KÖRPERSCHAFT DES ÖFFENTLICHEN RECHTS
VORSTAND: Prof. Dr. Claus-Dieter Heidecke (ÄV) Prof. Dr. Karlhans Endlich (WV) · Marie le Claire (KV)
AUFSICHTSRATSVORSITZENDER: Sebastian Schröder
Fleischmannstraße 8 · 17475 Greifswald · Tel.: +49 (0) 3834 86 0 · www.medizin.uni-greifswald.de

DIE DEUTSCHEN UNIVERSITÄTSKLINIKA®

correctly, Markov Blanket feature selection, such as described in Shen et al., is typically applied as a first step with the aim of improving predictive performance in the subsequent modeling step. Usually, algorithms are used that are similar to BN structure learning. We would like to mention, that in this respect, our approach differs from the above literature. Random forests perform feature selection internally, and the strategy differs from Markov Blanket Learning, especially because direct and indirect influences are not distinguished. Moreover, in our approach, the subsequent BN step aims to explain the interrelationships of the selected features, rather than to optimize performance. However, we use the concept of Markov Blankets for the interpretation and evaluation of our model. We, thus, added the recommended references and further explanations to the introduction (lines 131-135 as well as references 23-26) and we feel that the point well complements our manuscript.

2. *Please note in the Results and Discussion paper that all of the algorithm details are specified in the Methods section. While that's a reasonable place to expect those things to be, and while the level of detail in that section is adequate, the paper is sufficiently technical that I've asked myself what the RF- and BN-fitting details might be at least 20 times before finally reaching the methods section. It might even be worth it to invest a sentence per algorithm to give at least a high-level outline of the methods the first time they are mentioned in Results.*

Author response: We appreciate the reviewer's feedback. We now added more model definitions to the section in which we describe the workflow (lines 147 - 159), where we also now explicitly refer to the methods part. We hope that the changes increase the understandability.

> "We propose a workflow that complements a RF model with a BN analysis for the post-hoc interpretation of inferred global predictive patterns. The network analysis allows an interpretation that goes beyond a ranking by global feature importance. The workflow consists of three main steps (Fig 1) and starts with careful training of a RF model. RF is an ensemble learning algorithm that aggregates multiple single decision trees. In the first step, we perform a hyperparameter optimization of the main RF parameters using a nested cross-validation approach. Afterwards, relevant features are identified from the RF model using two different FI measures. Due to the high number of features included, we use a statistical mixture model approach to distinguish relevant from irrelevant features. The latter are modeled by a component around a FI of zero. This step is followed by BN structure learning among all relevant predictors. BNs are probabilistic graphical models. The BN is trained using a score-based approach. This last step yields an interpretable feature association model. For further details on the workflow, we refer the reader to the methods section."

UNIVERSITÄTSMEDIZIN GREIFSWALD · KÖRPERSCHAFT DES ÖFFENTLICHEN RECHTS
VORSTAND: Prof. Dr. Claus-Dieter Heidecke (ÄV) Prof. Dr. Karlhans Endlich (WV) · Marie le Claire (KV)
AUFSICHTSRATSVORSITZENDER: Sebastian Schröder
Fleischmannstraße 8 · 17475 Greifswald · Tel.: +49 (0) 3834 86 0 · www.medizin.uni-greifswald.de

DIE DEUTSCHEN UNIVERSITÄTSKLINIKA®
